# Comparison of two SVD-based color image compression schemes

**DOI:** 10.1371/journal.pone.0172746

**Published:** 2017-03-03

**Authors:** Ying Li, Musheng Wei, Fengxia Zhang, Jianli Zhao

**Affiliations:** 1 College of Mathematical Sciences, Liaocheng University, Shandong, P. R. China; 2 College of Mathematics and Science, Shanghai Normal University, Shanghai, P. R. China; University of Texas at San Antonio, UNITED STATES

## Abstract

Color image compression is a commonly used process to represent image data as few bits as possible, which removes redundancy in the data while maintaining an appropriate level of quality for the user. Color image compression algorithms based on quaternion are very common in recent years. In this paper, we propose a color image compression scheme, based on the real SVD, named real compression scheme. First, we form a new real rectangular matrix *C* according to the red, green and blue components of the original color image and perform the real SVD for *C*. Then we select several largest singular values and the corresponding vectors in the left and right unitary matrices to compress the color image. We compare the real compression scheme with quaternion compression scheme by performing quaternion SVD using the real structure-preserving algorithm. We compare the two schemes in terms of operation amount, assignment number, operation speed, PSNR and CR. The experimental results show that with the same numbers of selected singular values, the real compression scheme offers higher CR, much less operation time, but a little bit smaller PSNR than the quaternion compression scheme. When these two schemes have the same CR, the real compression scheme shows more prominent advantages both on the operation time and PSNR.

## 1 Introduction

An image is one of the most important information carriers involved in various areas such as medical imaging, defense, artwork, etc. The increasing use of digital images has raised the issue of storage and transmission. Compression is a commonly used process to represent image data as few bits as possible through exploiting redundancy in the data while maintaining an appropriate level of quality for the user [1–3]. Research on the technology of image compression began with digital TV signal in 1948. Over the past 70 years, a lot of gray-scale image compression method has been developed. However, due to rapid applications of the color image technique in the internet, researchers have turned their attention to color image compression [4, 5]. Compared with the gray-scale image, the color image can greatly improve the capacity and fidelity of the information. It can be broken into multiple prime color channels, so the color image compression is more challenging comparing to compression of single channel gray-scale images. There are various techniques available in literatures for color image like YCbCr, RGB, YIQ and HSI, among which, RGB is apparently the most popular space, because this channel format is the most natural for representing color in the real world. Each of the three channels R, G and B is highly correlated with the other two.

One of the typical algorithms for color image compression is multichannel compression. For example, in [4], a color image is first decomposed into single color channels which are treated independently, The essence of this approach is to treat gray-scale image. Because there are strong links between each channel of a color image, to do so, it can not reflect the connection between the channels very well. Algorithms based on quaternion are very common in recent years. A pixel of a color image can be viewed as a quaternion number. So a color image can be considered as a quaternion matrix. If a color pixel is processed in a holistic manner, then the relationship of spectrum between the color channels will run throughout the processes of operation and processing. The color information will not be lost. But we must notice that, these kind of algorithms require much more operations. In addition, we also can construct a new real matrix with the components of a color image. In order to achieve the purpose of color image compression, we only need to deal with the real matrix. To some extent, it is not only keep the connection between the color channels, but also fully control the computational complexity.

Compression algorithms utilizing the Singular Value Decomposition(SVD) became more and more popular [5–11]. In [5], authors converted the RGB image into YCbCr format and then compressed the image by applying the SVD to each of the three components, brightness Y, blue chrominance Cb and red chrominance Cr. In [7], authors proposed an image compression technique based on the SVD and DCT. In [9, 10], the whole image was divided into several equal size of sub-blocks and the SVD was implemented on each sub-block. In [11], authors proposed a algorithm automatically select an appropriate number of singular values on each sub-block by studying the relationship between the required PSNR value and the number of singular values.

In this paper, we will propose the real SVD-based color compression scheme, named real compression scheme. We will compare this scheme with quaternion SVD-based scheme. In order to improve the speed of quaternion SVD, we adopt the real structure-preserving algorithm [12, 13]. We will compare the visual and quantitative results of these two color image compression schemes such as PSNR, CR, storage and computing speed. The rest of this paper is organized as follows. In Section 2, we present a brief overview regarding the SVD and quaternion representation of a color image. In Section 3, we introduce two kinds of SVD algorithms and compare their computing speed. In Section 4, we introduce two SVD-based color image compression scheme. The experimental results and analysis on speed, compression effect are presented in Section 5. Finally, our conclusion are stated in Section 6.

## 2 Priliminary

Throughout this paper, Let ℝ be the real number field, ℚ the quaternion skew-field. For any matrix *A*, *A*^*T*^ and *A*^*H*^ represent the transpose and conjugate transpose of *A*, respectively. ${\mathbb{R}}^{m\times n}$ and ${\mathbb{Q}}^{m\times n}$ are the set of all *m* × *n* real matrices and the set of all *m* × *n* quaternion matrices. *A*(:, *s*), *A*(:, *s*:*t*) denote all the rows, the *s*th column and the columns from the *s*th to the *t*th of matrix *A*, respectively.

### 2.1 Overview of the SVD

The SVD [14] is one of the most powerful tools in numerical algebra and is widely applied to digital image processing [7].

The SVD of an *m* × *n* matrix *A* is expressed as

$$\begin{array}{c} A=U\Sigma V^{H} \end{array}$$

where both *U*_*m* × *m*_ and *V*_*n* × *n*_ are unitary matrices and

$$\begin{array}{c} \Sigma =\left[\begin{array}{cc} \Sigma_{1} & 0\\ 0 & 0 \end{array}\right], \end{array}$$

Σ_1_ = *diag*(*σ*_1_, *σ*_2_, …, *σ*_*r*_), *σ*_1_ ≥ *σ*_2_ ≥ … ≥ *σ*_*r*_ > 0 are positive singular values of *A*, and *r* is the rank of matrix *A*. If *A* is a real matrix, then both *U*_*m* × *m*_ and *V*_*n* × *n*_ are real orthogonal matrices and

$$\begin{array}{c} A=U\Sigma V^{T}. \end{array}$$

The SVD of a quaternion matrix was theoretically derived in 1997 by Zhang [15]. Now the SVD of a quaternion matrix is also widely used in color image processing. The main advantage of the SVD is that it localizes most of the energy content of the matrix into few singular values. As *σ*_1_ is the maximum value, its significance in describing image feature is highest. Values of the diagonal elements of Σ are decrease, and so also their significance in describing image features. The singular values of the matrix decrease quickly with the increasing rank. We can compress the matrix data by eliminating the small singular values with this property, and it will not significantly reduce the visual quality of the image.

### 2.2 Quaternion representation of a color image

First, we recall some basic properties about quaternion and quaternion matrices.

A quaternion q∈ℚ is constituted of one real part and three imaginary parts

$$\begin{array}{c} q=a+bi+cj+dk, \end{array}$$

where a,b,c,d∈ℝ, and three imaginary units *i*, *j*, *k* satisfy

$$\begin{array}{c} i^{2}=j^{2}=k^{2}=-1,\;\;ij=-ji=k,\;\;ki=-ik=j,\;\;jk=-kj=i. \end{array}$$

When the real part *a* = 0, *q* is called pure imaginary quaternion. Quaternion are an extension of complex numbers from 2D plane to the 3D space or 4D space. The quaternion skew-field ℚ is an associative but non-commutative algebra of rank four over ℝ. Many scholars have carried on thorough and careful research in the quaternion theoretical area. For example, [12, 13, 16–20] discussed several different kinds of problems of quaternion matrices such as algorithms for qLS problem, Householder based transformations and qSVD, quaternion Hermitian eigenvalue problems, quaternion LU decomposition, quaternion matrix equations and etc.

In [21], Sangwine proposed to encode the three channel components of a RGB image on the three imaginary parts of a pure imaginary quaternion, that is

$$\begin{array}{c} q(x,y)=r(x,y)i+g(x,y)j+b(x,y)k, \end{array}$$

where *r*(*x*, *y*), *g*(*x*, *y*) and *b*(*x*, *y*) are the red, green and blue components of the pixel at position (*x*, *y*), respectively. Thus, a color image with *m* rows and *n* columns can be represented by a pure imaginary quaternion matrix

$$\begin{array}{c} A={\left(q_{ij}\right)}_{m\times n}=Ri+Gj+Bk,\;\;q_{ij}\in Q. \end{array}$$

Since then, quaternion representation of color image has attracted great attention. Many researchers applied the quaternion matrix to study the problems of color image processing due to its capability to treat the three color channels holistically without losing color information.

## 3 Comparison of real SVD and quaternion SVD

For a quaternion matrix $A\in {\mathbb{Q}}^{m\times n}$ of a color image, we can perform the quaternion SVD with different kinds of algorithms. For example, in [22], authors provided the function ‘svd’ in Matlab Toolbox using quaternion arithmetics. In [12, 13], authors proposed a real structure-preserving algorithm based on the following results.

For any quaternion matrix *A* = *A*_1_ + *A*_2_*i* + *A*_3_*j* + *A*_4_*k*, where $A_{1},A_{2},A_{3},A_{4}\in {\mathbb{R}}^{m\times n}$, its real representation can be defined as follows [12, 13],

$$\begin{array}{c} A^{R}\equiv \left[\begin{array}{cccc} A_{1} & -A_{2} & -A_{3} & -A_{4}\\ A_{2} & A_{1} & -A_{4} & A_{3}\\ A_{3} & A_{4} & A_{1} & -A_{2}\\ A_{4} & -A_{3} & A_{2} & A_{1} \end{array}\right]. \end{array}$$ (3.1)

The properties of *A*^*R*^ are as follows.

**Theorem 3.1**. [23] *Let* $A,B\in {\mathbb{Q}}^{m\times n}$, $C\in {\mathbb{Q}}^{n\times s}$ *and* a∈ℝ. *Then*

1. (*A* + *B*)^*R*^ = *A*^*R*^ + *B*^*R*^, (*aA*)^*R*^ = *aA*^*R*^, (*AC*)^*R*^ = *A*^*R*^*C*^*R*^.
2. (*A*^*H*^)^*R*^ = (*A*^*R*^)^*T*^.
3. $A\in {\mathbb{Q}}^{m\times m}$ *is a unitary matrix if and only if A^R^ is an orthogonal matrix*.

From (3.1) and Theorem 3.1, for the matrix *A*^*R*^, we only need to store the first column block of *A*^*R*^, denoted as $A_{c}^{R}=\left[\begin{array}{c} A_{1}\\ A_{2}\\ A_{3}\\ A_{4} \end{array}\right].$

From this notation and Theorem 3.1, we have the following result.

**THEOREM 3.2**. *Let* $A,B\in {\mathbb{Q}}^{m\times n}$, $C\in {\mathbb{Q}}^{n\times s}$, $q\in {\mathbb{Q}}^{m}$ *and* a∈ℝ. *Then*

1. ${\left(A+B\right)}_{c}^{R}=A_{c}^{R}+B_{c}^{R},\;{\left(aA\right)}_{c}^{R}=aA_{c}^{R},\;{\left(AC\right)}_{c}^{R}=A^{R}C_{c}^{R}.$
2. ${\left(A^{H}\right)}_{c}^{R}={\left[{\left(A^{R}\right)}^{T}\right]}_{c}.$
3. ${∥A∥}_{F}={∥A_{c}^{R}∥}_{F},\;{∥q∥}_{2}={∥q_{c}^{R}∥}_{2}.$

In addition, we have the following results about the SVD of a quaternion matrix and its real representation.

**THEOREM 3.3**. *Let* $A\in {\mathbb{Q}}^{m\times n}$. *Then the singular values of A^R^ appear in fours*.

Therefore, if we want to compute the SVD of a *m* × *n* quaternion matrix *A*, then we can deal with the SVD of the 4*m* × 4*n* real representation matrix *A*^*R*^. In fact, under the orthogonal transformations, the real representation of a quaternion matrix have the standard form stated in the next theorem.

**THEOREM 3.4**. [12] *Suppose that* $A\in {\mathbb{Q}}^{m\times n}$ *and A^R^ is the real representation of A*. *Then there exist orthogonal matrices* $U\in {\mathbb{R}}^{4m\times 4m}$ and $V\in {\mathbb{R}}^{4n\times 4n}$ *such that*

$$\begin{array}{c} U^{T}A^{R}V=\left[\begin{array}{cccc} D & 0 & 0 & 0\\ 0 & D & 0 & 0\\ 0 & 0 & D & 0\\ 0 & 0 & 0 & D \end{array}\right], \end{array}$$ (3.2)

*where* $D\in {\mathbb{R}}^{m\times n}$ *is a bidiagonal matrix*.

From Theorem 3.3 and 3.4, the SVD of *A*^*R*^ can be obtained by computing the SVD of bidiagonal matrix *D*. So, we should first get bidiagonal matrix *D* using Householder based transformation. In [13], we listed three forms Householder based transformations appeared in literatures and proposed a new form of quaternion Householder based transformation. And then, we gave the real structure-preserving algorithms for these quaternion Householder based transformations. By comparison on computation amounts and assignment numbers, we obtained the most flexible and efficient one which is described as follows.

**THEOREM 3.5**. *Suppose that* $0\neq y\in {\mathbb{Q}}^{n}$ *is not a multiple of e*_1_, *denote* $u=\frac{y-\alpha e_{1}}{\left\|y-\alpha e_{1}\right\|}$, *where*

$$\begin{array}{c} \alpha =\left\{\begin{array}{cc} -\frac{y_{1}}{|y_{1}|}∥y∥,\; & y_{1}\neq 0,\\ -∥y∥,\; & otherwise, \end{array}\right. \end{array}$$

$\alpha_{M}=diag\left(\frac{\bar{\alpha }}{\left|\alpha \right|},I_{n-1}\right)$, *and H* = *α*_*M*_(*I* − 2*uu*^*H*^), *then H maps y to* |*α*|*e*_1_.

This kind of Householder based transformation is *H*4 introduced in [13]. Its specific real structure-preserving algorithm can refer to Algorithm 4.1, 4.2 and 4.8 in [13].

After the bidiagonal matrix *D* is calculated, we can perform a sequence of iterations using Givens rotations on it, then the SVD of quaternion matrix is obtained.

On the other hand, we can extract the three imaginary parts *R*, *G* and *B* of matrix *A* to rearrange as a new real rectangle matrix

$$\begin{array}{c} C=\left[\begin{array}{c} R\\ G\\ B \end{array}\right], \end{array}$$

then directly use the function ‘svd’ on the real matrix *C*.

**REMARK 3.1**. *In* Table 1 *we list the numbers of real flops and assignment numbers for computing real SVD for* $C\in {\mathbb{R}}^{3m\times n}$ *and quaternion SVD for* $A\in {\mathbb{Q}}^{m\times n}$ *using real structure-preserving algorithm*, *where assignment numbers refer to call subroutines or perform matrix operations*. *In matrix operations*, *say*, *B* = *AX* + *Y*, *we adopt the assignment B* = *A* * *X* + *Y to utilize vector pipelining arithmetic operations rather than explicitly using triply-nested for-end loops*, *to speed up computations remarkably*. *Therefore*, *real arithmetic numbers as well as assignment numbers are important measures*. *See*, *e*. *g*., *Charter* 1 *of* [14].

**Table 1:** Computation amounts and assignment numbers for real SVD and quaternion SVD.

	real flops	assignment number
real SVD	12*mn*^2^ + 9*n*^3^	6*n*
quaternion SVD	$96mn^{2}+\frac{64}{3}n^{3}$	10*n*

We now provide a numerical example to compare the efficiency of the three algorithms mentioned above. All these computation are performed on an Intel Core i5@ 2.20Ghz/8GB computer using MATLAB R2013a.

**Example 3.1**. *For k* = 1:50, *m* = 10 * *k*, $A\in {\mathbb{Q}}^{m\times m}$, *we apply the above three different algorithms to compute the SVD*. *We compare the CPU times of three algorithms*: *qSVD in toolbox for A* [22], *the structure-preserving SVD for A and the real SVD for C*.

**Fig 1:**
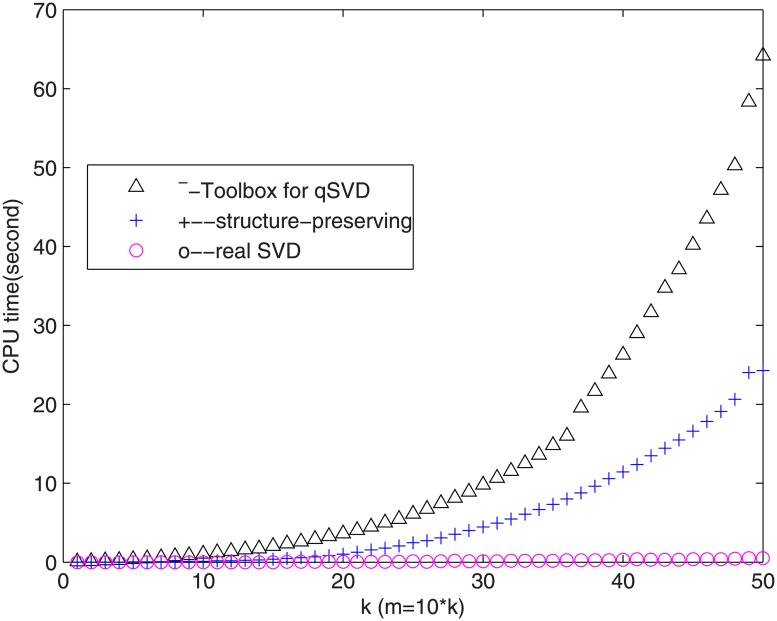
Comparison of the SVD algorithms.

In [12, 13], we have already shown that structure-preserving algorithm is superior to quaternion command ‘svd’ in Matlab. Fig 1 shows that the CPU time of the real SVD algorithm for *C* is the smallest. Especially, when the matrix is bigger, its superiority is more obvious.

From the above discussion and Fig 1, we see that the algorithm performing the SVD for *C* is most efficient. In next section, we will introduce two kinds of color image compression schemes based on the real SVD for *C* and quaternion SVD for *A*, respectively.

## 4 Two SVD-based color image compression schemes

In this section, we describe two SVD-based color image compression schemes. Assume that original image *A* is a RGB color image of size *N* × *N*.

### 4.1 The real compression scheme

We can form a new real rectangle matrix composed by the red, green and blue components of color image, and accordingly we obtain the real compression scheme. The detail is described as follows.

*Step*1. Pre-processing on color image compression

First, the original RGB color image *A* is broken into its different constituent components by dimension reduction treatment. Then we obtain the 2-D component matrices R, G and B. Next, the R, G and B color components matrices are rearranged as a rectangle matrix $C=\left[\begin{array}{c} R\\ G\\ B \end{array}\right]$ of 3*N* × *N*.

*Step*2. Performing real SVD for *C*

We perform the real SVD for *C*: $C=U_{C}\Sigma_{C}V_{C}^{T},$ where Σ_*C*_ = diag(*σ*_1_, ⋯, *σ*_*N*_).

*Step*3. Color image compressing

Assume *q* is a integer and *q* < *N*, denote

$$\begin{array}{c} C_{C}=U_{C}\left(:,1:q\right)\Sigma_{C}\left(1:q,1:q\right)V_{C}{\left(:,1:q\right)}^{T}. \end{array}$$

By removing the small singular value data, we get a new matrix *C*_*C*_. We can extract the corresponding rows as the R, G and B components and form a 3-D color image again. Then a compressed image is constructed.

### 4.2 The quaternion compression scheme

Except for the real compression scheme above mentioned, we can reconstruct a compressed color image by implementing quaternion SVD for *A* using real structure-preserving algorithm as follows.

*Step*1. Performing quaternion SVD for *A* using real structure-preserving algorithm

First, we form the real representation of *A*

$$\begin{array}{c} A^{R}\equiv \left[\begin{array}{cccc} 0 & -R & -G & -B\\ R & 0 & -B & G\\ G & B & 0 & -R\\ B & -G & R & 0 \end{array}\right] \end{array}$$ (4.1)

and then take out the first column block of *A*^*R*^, denoted as $B=\left[\begin{array}{c} 0\\ R\\ G\\ B \end{array}\right].$ We perform real structure-preserving algorithm to get the SVD of *A*: $A=U_{A}\Sigma_{A}V_{A}^{T},$ where Σ_*A*_ = diag(*σ*_*A*_1__, ⋯, *σ*_*A*_*N*__).

*Step*2. Color image compressing

Assume *q* is a integer and *q* < *N*, denote

$$\begin{array}{c} A_{C}=U_{A}\left(:,1:q\right)\Sigma_{A}\left(1:q,1:q\right)V_{A}{\left(:,1:q\right)}^{H}. \end{array}$$

By removing the small singular value data, we get a new quaternion matrix *A*_*C*_. Since the quaternion matrix of a color image is pure imaginary, we modify the real part of *A*_*C*_ as zero matrix to get a pure imaginary quaternion matrix $\tilde{A_{C}}$, which is corresponding to the compressed color image.

## 5 Comparison of two color image compression schemes

In this section, we carry out four experiments with different original images [24] to evaluate the effectiveness of the above two compression schemes. All the computation are performed on an Intel Core i5@ 2.20Ghz/8GB computer using MATLAB R2013a.

**Fig 2:**
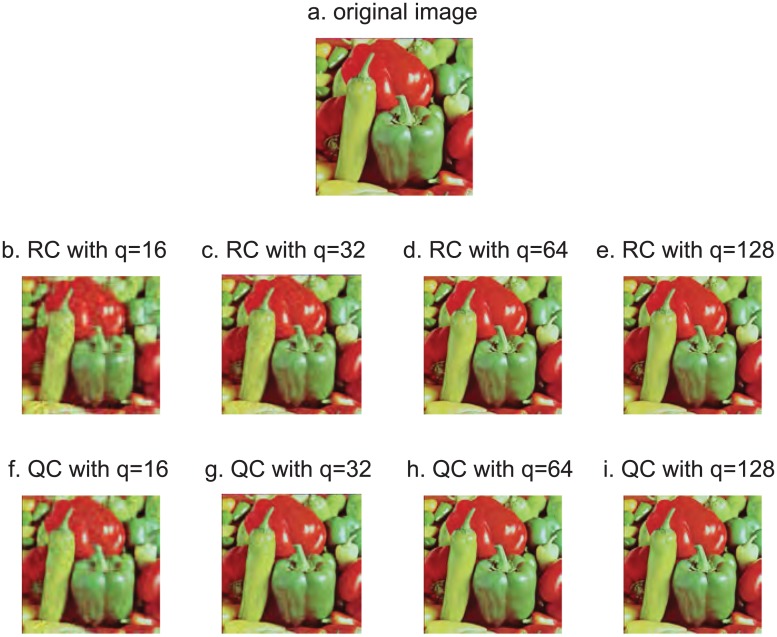
Original and compressed image Pepper using two schemes with different *q*.

**Example 5.1**. *Color image Pepper of size* 512 × 512 *is taken as the original image*, *shown in* (*a*) *of* Fig 2. *The compressed images with q* = 16, 32, 64, 128 *using real compression scheme are shown in* (*b*) − (*e*) *of* Fig 2. *The corresponding images are shown in* (*f*) − (*i*) *of* Fig 2 *using quaternion compression scheme*.

From Fig 2, we observe that the visual effect of compressed images are similar with the same *q* in these two compression schemes. We will use several different quantitative criteria to judge the effect of compression algorithm, PSNR, CR and speed.

The visual fidelity can be measured by calculating two types of parameters known as Mean Square Error(MSE) or Peak Signal to Noise Ratio(PSNR) [25, 26] between the original image *A* and the compressed image $\tilde{A}.$ Mean Square Error(MSE) is defined as

$$\begin{array}{c} MSE=\frac{\sum_{x=1}^{N}\sum_{y=1}^{N}\sum_{k=1}^{3}{\left(A\left(x,y,k\right)-\tilde{A}\left(x,y,k\right)\right)}^{2}}{3N^{2}}. \end{array}$$

and Peak Signal to Noise Ratio (PSNR) is defined as

$$\begin{array}{c} PSNR=10lg\frac{3N^{2}{\left(\max A(x,y,k)\right)}^{2}}{\sum_{x=1}^{N}\sum_{y=1}^{N}\sum_{k=1}^{3}{\left(A\left(x,y,k\right)-\tilde{A}\left(x,y,k\right)\right)}^{2}} \end{array}$$

where *A* is a matrix of size *N* × *N*, max *A*(*x*, *y*, *k*) represents the maximum pixel value of a color image, and here it is 255. *A*(*x*, *y*, *k*) and $\tilde{A}(x,y,k)$ are the pixel values location at position (*x*, *y*, *k*) in the original image and the compressed image, respectively. In general, the lower MSE or the larger PSNR means better image quality.

Compression Ratio(CR) is a term that is being used to describe ratio of compressed image to the original image defined as

$$\begin{array}{c} CR=\frac{N^{2}}{K}, \end{array}$$

where *K* is the size of compressed image. We can use CR to judge how compression efficiency is, as higher CR means better compression.

Compression speed is influenced by computational complexity and size of storage. Computational complexity is an important factor that any commercial entity would take into consideration, particularly if the volume of images is significant.

Table 2 is showing the quantitative comparison and compression ratio of the two techniques with different *q*, the numbers of selected singular values. In all the following Tables, PSNR1, SIZE1, CR1 and CPU1 stand for the corresponding values of real compression scheme, PSNR2, SIZE2, CR2 and CPU2 stand for the corresponding values of quaternion compression scheme.

**Table 2:** PSNR and CR values with different numbers of selected singular values.

image	N	q	PSNR1	PSNR2	CR1	CR2
Pepper	512	8	14.5384	15.4628	48	24
Pepper	512	16	17.6304	18.6249	24	12
Pepper	512	32	21.0140	21.8394	12	6
Pepper	512	64	24.8774	24.8440	6	3
Pepper	512	128	28.8143	27.2391	3	1.5

From Table 2, we observe PSNR values obtained by these two schemes have little difference when the numbers of selected singular values are same, while the real scheme is much faster than the quaternion one. This is one of the advantages of real compression scheme. In fact, assume that the SVD of *C* is as described in step 2 of real compression scheme in section 4, and the SVD of *A*^*R*^ is as follows

$$\begin{array}{c} A^{R}=U_{A^{R}}\Sigma_{A^{R}}V_{A^{R}}^{T}. \end{array}$$ (5.1)

Assume that the number of selected singular values are all *q*. Since $C\in {\mathbb{R}}^{3N\times N},$ we should store $U_{C}\in {\mathbb{R}}^{3N\times q}$, diagonal matrix $\Sigma_{C}\in {\mathbb{R}}^{q\times q},$ and $V_{C}\in {\mathbb{R}}^{N\times q}$ in the SVD of *C*, the storage is 4*Nq* + *q*, and $CR1=\frac{3N^{2}}{4Nq+q}\approx \frac{3N}{4q}.$ In the real structure-preserving algorithm, the first column block, *B*, of *A*^*R*^ is a 4*N* × *N* matrix. Since the real part of *A* is zero, then the storage of *A*^*R*^ is also 3*N*^2^. While the storage is 8*Nq* + *q* in the SVD of *A*^*R*^ in Eq (5.1), which is much bigger than that of SVD of *C*. Then we obtain $CR2=\frac{3N^{2}}{8Nq+q}\approx \frac{3N}{8q}$. That is, *CR*1 is twice as large as *CR*2. Under the condition of same storage, the number of selected singular values of *C* can be twice as large as that of *A* to ensure good compression effect. The comparison of compression effect of two different schemes with the same storage will be presented in Table 3.

**Table 3:** PSNR and CPU time with the same CR.

image	N	PSNR1	PSNR2	CPU1(S)	CPU2(S)
Lena	256	19.0967	17.2677	0.0743	2.7355
Lena	256	22.6707	20.0926	0.0791	2.7715
Lena	256	27.8540	23.7107	0.0812	2.7721
Pepper	512	17.6304	15.4628	0.4594	24.6182
Pepper	512	21.0140	18.6249	0.4561	25.2941
Pepper	512	24.8774	21.8394	0.4520	24.7409
Pepper	512	28.8143	24.8440	0.4530	24.9629
Butterfly	1024	21.3900	18.2636	3.2957	211.9598
Butterfly	1024	25.7975	21.5778	3.3670	210.2415
Butterfly	1024	30.8166	26.1665	3.3929	216.2491
Butterfly	1024	36.0006	31.6080	3.3342	212.8359
Butterfly	2048	21.3805	18.2589	24.8641	1641.1
Butterfly	2048	25.7771	21.5676	25.3141	1694.6
Butterfly	2048	30.7727	26.1443	26.3486	1637.0
Butterfly	2048	35.9325	31.5601	26.6198	1744.1
Butterfly	2048	46.3690	38.5951	28.3470	1669.3

Assume the SVD of quaternion matrix *A* of color image Pepper is

$$\begin{array}{c} A=U_{A}\Sigma_{A}V_{A}^{T}. \end{array}$$ (5.2)

**Fig 3:**
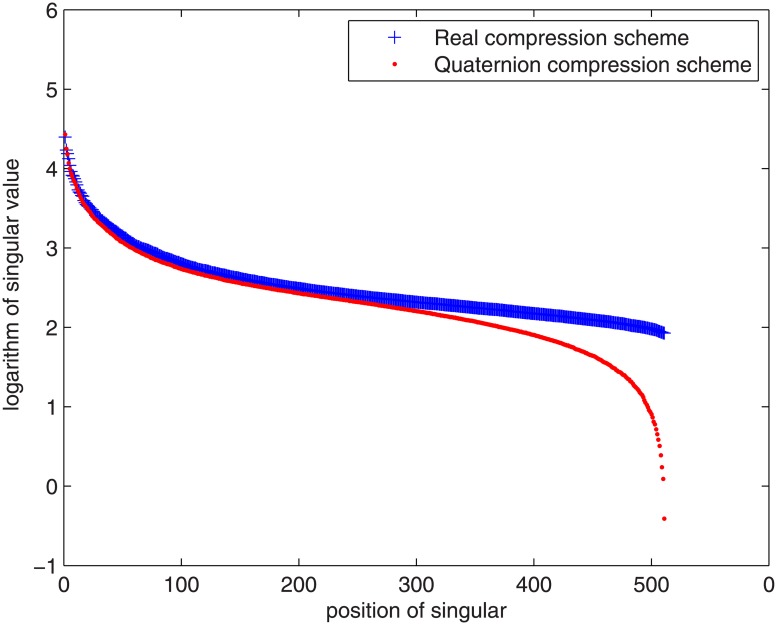
Comparison of singular values of *C* and *A* of 512 × 512 image.

In Fig 3, we compare the singular values of *C* and *A*. Because there is wide gap between the order of magnitude of the largest and smallest singular values, In order to facilitate comparison, we perform logarithmic transformation for all the singular values. As shown in Fig 3, we observe that the several largest singular values of *A* are larger than those of *C*. But the most smaller singular values of *A* are smaller than those of *C*. The reason is as follows.

We know, one of the most frequently used matrix norm in numerical linear algebra is the Frobenius norm, for a *m* × *n* matrix *A*, defined as

$$\begin{array}{c} {∥A∥}_{F}=\sqrt{\sum_{i=1}^{m}\sum_{j=1}^{n}{\left|a_{ij}\right|}^{2}}. \end{array}$$

It has the following connections to the SVD of *A*,

$$\begin{array}{c} {∥A∥}_{F}=\sigma_{1}^{2}+\sigma_{2}^{2}+\cdots +\sigma_{p}^{2},\;\;p=\min\left\{m,n\right\}. \end{array}$$

For any quaternion matrix *A* = *A*_1_ + *A*_2_*i* + *A*_3_*j* + *A*_4_*k*, it is not difficult to verify that

$$\begin{array}{c} {∥A∥}_{F}^{2}=∥A_{1}{∥}_{F}^{2}+∥A_{2}{∥}_{F}^{2}+∥A_{3}{∥}_{F}^{2}+{∥A_{4}∥}_{F}^{2}, \end{array}$$

then for a given pure imaginary quaternion matrix *A* of a color image, we have

$$\begin{array}{c} {∥A∥}_{F}^{2}={∥R∥}_{F}^{2}+{∥G∥}_{F}^{2}+{∥B∥}_{F}^{2}. \end{array}$$

On the other hand, for the real rectangle matrix *C*, we also have

$$\begin{array}{c} {∥C∥}_{F}^{2}={∥R∥}_{F}^{2}+{∥G∥}_{F}^{2}+{∥B∥}_{F}^{2}, \end{array}$$

so

$$\begin{array}{c} {∥A∥}_{F}^{2}={∥C∥}_{F}^{2}, \end{array}$$

i.e. the sum of square of all the singular values of *A* is equal to that of *C*. The order of magnitude of the largest singular values is the fifth or fourth power of ten, thus the gap of these singular values of *A* and *C* is very large. This lead to the most smaller singular values of *A* are smaller than those of *C*. When we select the same numbers of the singular values in the two algorithms, it seems that the performance of the quaternion compression scheme is better than the real compression scheme. But we notice that when using the quaternion compression scheme, the real part of the reconstructed quaternion matrix may not be zero matrix. We should set it to be zero. In this step, the error inevitably increase. Thus we see the approximate results of the two schemes are similar. But the advantage of the real compression scheme in speed is obvious.

**Example 5.2**. *Color image Lena of* 256 × 256 *is taken as the original image*. *We provide the PSNR*, *CPU time with different numbers of selected singular values using the real compression scheme and quaternion compression scheme in* Table 4, *respectively*.

**Table 4:** PSNR and CPU time with the same number of selected singular values.

image	N	q	PSNR1	PSNR2	CPU1(S)	CPU2(S)
Lena	256	8	16.5426	17.2677	0.0691	2.7355
Lena	256	16	19.0967	20.0926	0.0743	2.7715
Lena	256	32	22.6707	23.7107	0.0791	2.7721
Lena	256	64	27.8540	29.2039	0.0812	2.7770
Butterfly	1024	16	18.1489	18.2636	3.1946	211.9598
Butterfly	1024	32	21.3900	21.5778	3.2957	210.2415
Butterfly	1024	64	25.7975	26.1665	3.3670	216.2491
Butterfly	1024	128	30.8166	31.6080	3.3929	212.8359
Butterfly	1024	256	36.0006	38.6748	3.3342	214.9876
Butterfly	2048	16	18.1443	18.2589	25.2482	1641.1
Butterfly	2048	32	21.3805	21.5676	24.8641	1694.6
Butterfly	2048	64	25.7771	26.1443	25.3141	1637.0
Butterfly	2048	128	30.7727	31.5601	26.3486	1744.1
Butterfly	2048	256	35.9325	38.5951	26.6198	1669.3
Butterfly	2048	512	46.3690	46.3690	28.3470	1694.2

In Example 5.3 and 5.4, we use 1024 × 1024 and 2048 × 2048 color image Butterfly as the original image, respectively, which are obtained by magnifying original image of 512 × 512 using the command ‘imresize’ in Matlab. The PSNR and CPU time in these two examples are all shown in Table 4. From Table 4, we observe the compression result in Example 5.2–5.4 consistent with Example 5.1. The PSNR of real compression scheme are a little bit smaller than those of quaternion one. This situation don’t change with the adjustment of image pixel. But as the image pixel is higher and higher, the gap of CPU time is becoming more and more big. Computation time of quaternion can reach 60 to 70 times of real compression scheme. Since speed is vital for problem of large image processing, it highlight the advantages of the real compression scheme.

Particularly, we compare the PSNR and computation time under the condition of the same compression ratio, i.e., the number of selected singular values of real compression scheme are twice of that of quaternion scheme. As shown in Table 3, the advantage of real compression scheme is not only reflected on the speed, the values of PSNR are much higher than those of quaternion compression scheme.

## 6 Conclusions

In this paper, we proposed a real SVD-based color image compression scheme. We formed a new real rectangle matrix *C* according to the red, green and blue components of the original color image and performed the real SVD for *C*. Then we selected several largest singular values and the corresponding vectors in the left and right unitary matrices to compress the color image. At the other hand, we viewed a color image as a quaternion matrix *A*. We performed quaternion SVD on *A* using the real structure-preserving algorithm to compress the original color image. We compared the two SVD-based color image compression schemes in terms of operation amount, assignment number and operation speed. We also compared their PSNR and CR. From numerical examples provided in Section 5, we observed that real compression scheme is more efficient with comprehensive consideration of various factors.

## References

[1] Wern CL, Ang LM, Phooi SK, Survey of image compression algorithms in wireless sensor networks. IEEE information technology, ITSim, International symposium. 2008;4:1–9.

[2] Eldin HZ, Elhosseini MA, Ali HA, Image compression algorithms in wireless multimedia sensor networks: A survey. Ain Shams Engineering Journal. 2015;6:481–490. 10.1016/j.asej.2014.11.001

[3] Ma T, Hempel M, Peng D, Sharif H, A survey of energy efficient compression and communication techniques for multimedia in resource constrained systems. Commun Surveys Tutorials, IEEE, 2013:963–972. 10.1109/SURV.2012.060912.00149

[4] Wu P, Xie K, Yu H, Zheng Y, Yu W, A new preprocessing algorithm used in color image compression. Advances in FCCS, 1, AISC. 2012;159:465–471.

[5] Singh SK, Kumar S, A framework to design novel SVD based color image compression. Third UKSim European Symposium on Computer Modeling and Simulation, IEEE. 2009: 235–240.

[6] Prasantha HS, Shashidhara HL, Murthy KNB, Image compression using SVD. International Conference on Computational Intelligence and Multimedia Applications. 2007.

[7] Andrews HC, Patterson CL, Singular value decompositions and digital image processing. IEEE Transactions on Acoustics, Speech and Signal Processing. 1976; 1(24):26–53. 10.1109/TASSP.1976.1162766

[8] Rufai AM, Anbarjafari G, Demirel H, Lossy image compression using singular value decomposition and wavelet difference reduction. Digital Signal Procossing. 2014;24:117–123. 10.1016/j.dsp.2013.09.008

[9] Aharon M, Elad M, Bruckstein A, K-SVD: an algorithm for designing overcomplete dictionaries for sparse representation. IEEE Transactions on Signal Processing. 2006;11(54):4311–4322. 10.1109/TSP.2006.881199

[10] Bryta O, Elad M, Compression of facialimages using the K-SVD algorithm. J. Visual Commmuion and Image Representation, 2008;19(4):270–282. 10.1016/j.jvcir.2008.03.001

[11] Shih YT, Chien CS, Chuang CY, An adaptive parameterized block-based singular value decomposition for image de-noise and compression. Appl. Math. Comput. 2012;218:10370–10385.

[12] Li Y, Wei M, Zhang F, Zhao J, A fast structure-preserving method for computing the singular value decomposition of quaternion matrix. Appl. Math. Comput. 2014;235:157–167.

[13] Li Y, Wei M, Zhang F, Zhao J, Real structure-preserving algorithms of Householder based transformations for quaternion matrices. J. Comput. Appl. Math. 2016;305:82–91. 10.1016/j.cam.2016.03.031

[14] Golub GH, Van Loan CF, Matrix Computations 4th Edition The Johns Hopkins University Press, Baltimore, MD 2013.

[15] Zhang F, Quaternions and matrices of quaternions. Linear Algebra Appl. 1997;251:21–57. 10.1016/0024-3795(95)00543-9

[16] Jiang T, Chen L, Algebraic algorithms for least squares problem in quaternionic quantum theory. Comput. Phys. Comm. 2007;176:481–485. 10.1016/j.cpc.2006.12.005

[17] Jiang T, Chen L, An algebraic method for Schroinger equations in quaternionic quantum mechanics. Comput. Phys. Comm. 2008;178:795–799. 10.1016/j.cpc.2008.01.038

[18] Wang M, Ma W, A structure-preserving for the quaternion LU decomposition in quaternionic quatumn theory. Comput. phys. Comm. 2013;184:2182–2186. 10.1016/j.cpc.2013.05.001

[19] Wang M, Wei M, Yang F, An iterative algorithm for least squares problem in quaternionic quantum theory. Comput. Phys. Comm. 2008;179:203–207. 10.1016/j.cpc.2008.02.016

[20] Jia Z, Wei M, Ling S, A new structure-preserving method for quaternion Hermitian eigenvalue problems. J. Comput. Appl. Math. 2013;239:12–24. 10.1016/j.cam.2012.09.018

[21] Sangwine SJ, Fourier transforms of colour images using quaternion, or hypercomplex numbers. Electron. Lett. 1996;21:1979–1980.

[22] Sangwine SJ, Bihan NL, Quaternion toolbox for matlab. http://qtfm.sourceforge.net/.

[23] Wang M, Ma W, A structure-preserving algorithm for the quaternion Cholesky decomposition. Appl. Math. Comput. 2013;223:354–361.

[24] Gonzalez RC, Woods RE, Digital Image Processing(2nd Edition). Bosston: Addison-Wesley Longman Publishing Co., Inc 2001.

[25] Ghorbel O, Ayedi W, Jmal MW, Abid M, Image compression in WSN: performance analysis. In: Communication technology(ICCT), IEEE 14th international conference.2012: 1363–1368.

[26] Kumar V, Kumar A, Bhardwaj A, Performance evaluation for image compression techniques. In: Devices, circuits and systems(ICDCS) international conference, 2012: 447–450.

